# Co-detection of respiratory pathogens among ILI patients: characterization of samples collected during the 2018/19 and 2019/20 pre-pandemic seasons

**DOI:** 10.1186/s12879-024-09687-1

**Published:** 2024-08-29

**Authors:** Allegra Ferrari, Irene Schiavetti, Matilde Ogliastro, Carola Minet, Raffaella Sibilio, Irene Giberti, Elisabetta Costa, Elvira Massaro, Piero Luigi Lai, Stefano Mosca, Bianca Bruzzone, Andrea Orsi, Donatella Panatto, Giancarlo Icardi

**Affiliations:** 1 Department of Health Sciences (DISSAL), University of Genoa, Genoa, Italy; 2 Interuniversity Research Center On Influenza and Other Transmissible Infections (CIRI-IT), Genoa, Italy; 3 IRCCS Ospedale Policlinico San Martino, Genoa, Italy

**Keywords:** Co-infection, Respiratory tract infection, Respiratory pathogens, LRI, URI, Syndromic surveillance

## Abstract

Influenza-like illness (ILI) patients co-detected with respiratory pathogens exhibit poorer health outcomes than those with single infections. To address the paucity of knowledge concerning the incidence of concurrent respiratory pathogens, their relationships, and the clinical differences between patients detected with single and multiple pathogens, we performed an in-depth characterization of the oropharyngeal samples of primary care patients collected in Genoa (Northwest Italy), during winter seasons 2018/19–2019/20.

The *apriori* algorithm was employed to evaluate the incidence of viral, bacterial, and viral-bacterial pairs during the study period. The grade of correlation between pathogens was investigated using the Phi coefficient. Factors associated with viral, bacterial or viral-bacterial co-detection were assessed using logistic regression.

The most frequently identified pathogens included influenza A, rhinovirus*, Haemophilus influenzae* and *Streptococcus pneumoniae.* The highest correlations were found between bacterial-bacterial and viral-bacterial pairs, such as *Haemophilus influenzae-Streptococcus pneumoniae*, adenovirus*-Haemophilus influenzae*, adenovirus*-Streptococcus pneumoniae*, RSV-A*-Bordetella pertussis*, and influenza B Victoria*-Bordetella parapertussis*. Viruses were detected together at significantly lower rates. Notably, rhinovirus, influenza, and RSV exhibited significant negative correlations with each other. Co-detection was more prevalent in children aged < 4, and cough was shown to be a reliable indicator of viral co-detection.

Given the evolving epidemiological landscape following the COVID-19 pandemic, future research utilizing the methodology described here, while considering the circulation of SARS-CoV-2, could further enrich the understanding of concurrent respiratory pathogens.

**Supplementary Information:**

The online version contains supplementary material available at 10.1186/s12879-024-09687-1.

## Background

According to the Global Burden of Disease (GBD) collaboration, globally, in 2019 there were about 17.2 billion cases of upper respiratory tract infection (URI) and 489 million cases of lower respiratory tract infection (LRI), which lead to, respectively, 10,000 and 2.5 million deaths. The highest incidence and mortality rates are observed among elders and children younger than 5 years, those who are immunocompromised or have underlying comorbidities such as chronic heart and respiratory disease [1].

On the other end of the spectrum of URIs, influenza-like illness (ILI) is distinguished by the sudden onset of at least one among four systemic symptoms (fever or feverishness, malaise, headache, myalgia) and one among three respiratory symptoms (cough, sore throat, shortness of breath). In the US, during the 2020–21 season, approximately 3000 health care providers reported an average 85 million outpatient visits for ILI symptoms [2].

With the development of multiplex PCR assays, which can identify numerous pathogens in a single sample, it has been found that many patients (20–40%) who present with ILI are actually infected with multiple viruses [3].

By both damaging the airway epithelium and dysregulating the inflammatory responses, viruses also predispose to secondary bacterial infection throughout the respiratory tract.

Multiple additional infections have been observed, among others, concomitantly to influenza A, influenza B, respiratory syncytial virus (RSV), rhinovirus, human coronavirus, parainfluenza virus and adenovirus. *Streptococcus pneumoniae, Haemophilus influenzae*, *Moraxella catarrhalis*, *Staphylococci* and respiratory anaerobes are predominant in both acute and chronic rhinosinusitis [4].

An analysis of 19,31 patients with respiratory infections has shown that, compared with other groups, patients with co-infections have both higher intensive care unit (ICU) admission and mortality rates, especially in case of laboratory-confirmed viral-bacterial co-infections [5].

Given the increased morbidity and mortality, the ability to identify co-infections represents an important advancement. However, there is currently a lack of knowledge regarding the relative frequency of co-detection of respiratory pathogens, their relationships, and the clinical differences between the presence of single and multiple pathogens.

This paucity of data is especially evident in the pre-COVID era, when limited research on co-detection of respiratory pathogens was carried out. As the current scientific discourse largely centers around SARS-CoV-2 co-infections, retrospective analyses can shed light on potential changes in the epidemiological patterns and their clinical implications, contributing to a more holistic comprehension of respiratory infections.

## Study objective

The scope of this study was to gather comprehensive data on the incidence of concurrent respiratory pathogens, their relationships, and the clinical differences between patients detected with single and multiple pathogens.

In order to do so, an in-depth characterization of the oropharyngeal samples of ILI patients attending primary care in Liguria (Northwest Italy), over the course of two pre-pandemic seasons (2018–19 and 2019–20), was performed in 3 steps:

1. Calculating the incidence of viral, bacterial, and viral-bacterial pairs of pathogens co-detected during the study period;
2. Establishing the grade of correlation between respiratory pathogens;
3. Identifying factors associated with the co-detection of viral, bacterial or viral-bacterial pathogens;

## Methods

### Study setting and population

Data was collected by the Interuniversity Research Center on Influenza and other Transmissible Infections (CIRI-IT) during winter seasons 2018–19 and 2019–20 within the framework of the DRIVE study. This was a European observational case–control study (design test-negative) meant to measure seasonal influenza vaccination effectiveness (IVE) against laboratory-confirmed influenza [6].

The study population consisted of patients aged 6 months and above, with no contraindication for influenza vaccination, who consulted a GPs or paediatrician who were part of the study network, for symptoms compatible with ILI in accordance with the ECDC case definition [7]. Inclusion and exclusion criteria are extensively described in the DRIVE protocol [8].

Demographic characteristics, chronic conditions and risk factors were collected by means of a standardized questionnaire. Subjects were enrolled from week 45/2018 to week 18/2019 and from week 44/2019 to week 11/2020.

The minimal required laboratory analyses for the DRIVE study included detection of influenza viruses and subtyping of positive samples. However, additional laboratory analyses were performed and used, retrospectively, for the completion of this manuscript.

### Laboratory analysis

In accordance with national protocols, pathogens in respiratory samples were identified empoying molecular assays.

The genetic material was extracted from each respiratory swab and set up for PCR with the Nimbus IVD Seegene platform (STARMag 96 × 4 Viral DNA/RNA Universal Kit).

Overall, the presence of 26 pathogens in the extracted material was investigated, through a one-step real-time multiplex retro-transcription RT-PCR assay on a Biorad CFX96™ thermal cycler. Three positive controls (one for each respiratory panel) and one internal control for viruses (common to all respiratory panels) were used for the analysis (included in the Seegene kit).

The kit used for the detection of 19 respiratory virus (RSV-A, RSV-B, influenza A(H1N1) and A(H3N2), influenza B, adenovirus, enterovirus, metapneumovirus, parainfluenza virus (PIV) 1, PIV-2, PIV-3, PIV-4, bocaviruses 1–4, rhinovirus and coronaviruses (229E, NL63, OC43)) and 7 bacteria pathogens (*Streptococcus pneumoniae, Bordetella parapertussis, Bordetella pertussis, Chlamydophila pneumoniae, Haemophilus influenzae, Legionella pneumophila, Mycoplasma pneumoniae*) was the AllplexTM Respiratory Panel Assays (https://adenovirus.seegene.com/assays/allplex_respiratory_panel_assays). Because this kit is not meant for the subtyping of influenza B, influenza B positive samples were characterized into B-Yamagata and B-Victoria lineages through a one-step real-time multiplex RT-PCR assay. To check extraction performance, amplification of the human ribonuclease P gene (RNP) was carried out at the same time; this procedure utilized a specific primer/probe set and adopted the same thermal profile as that of the influenza A/B virus real time RT-PCR assay [9].

Samples showing a cycle threshold (Ct) value < 40 were considered positive. Sample aliquots were stored at -20˚C in order to be used for future studies. Indeed, because participants of the DRIVE study were enrolled until week 11 of 2020, preservation of aliquots allowed us to later test all samples for SARS-CoV-2, in order to assess a possible premature circulation of the virus. For the detection of SARS-CoV-2, the AllplexTM SARS-CoV-2 assay, able to detect 4 genes of the virus, was employed (https://adenovirus.seegene.com/assays/allplex_sars_cov_2_assay).

### Statistical analysis

The incidence of each respiratory pathogen, as well as pairs of viral, bacterial, and viral-bacterial pathogens during the study period, was evaluated using the *apriori* algorithm.

Unlike conventional incidence ratios, in the context of complex interactions within the dataset, the use of the *apriori* algorithm allowed us to consider scenarios where pathogens are not only detected in pairs, but also as part of more numerous pathogen combinations. For instance, when examining the incidence of the *Streptococcus pneumoniae* and *Haemophilus influenzae* pair, the algorithm allowed us to calculate its incidence when these pathogens are found not only alone (a sample positive to *Streptococcus pneumoniae* and *Haemophilus influenza*), but within larger sets of pathogens (eg. a sample positive to *Streptococcus pneumoniae*, *Haemophilus influenzae* and influenza A or *Streptococcus pneumoniae*, *Haemophilus influenzae,* influenza A and adenovirus).

Specifically, this process involves first identifying frequently appearing individual pathogens (individual “items” with a frequency greater than or equal to a given “support” threshold) within the dataset. Subsequently, the algorithm systematically extends these individual findings into broader, frequent sets of pathogen combinations (“frequent itemsets”) [10]. The algorithm was implemented using KNIME Analytics Platform version 4.6.0 (University of Konstanz, Zurich, Switzerland).

**Table:** 

Example of algorithm output in the context of our dataset:	
○ itemset: adenovirus, rhinovirus (pathogen pair of interest) ○ itemset size: 2 (number of items, in this case pathogens, contained in the itemset) ○ itemset support: 29 (number of times the itemset is contained in the database, alone or in association with other items) ○ relative itemset support: 1.43% (rate at which the itemset is contained in the database, alone or in association with other items)	
The “relative itemset support” corresponds to the incidence rate at which an itemset (a pathogen or pair of pathogens) is contained in the database, alone or in association with other items, during the study period	

The grade of correlation between different viral and bacterial respiratory pathogens was investigated using the Phi coefficient.

Differences in continuous variables between two seasons were investigated with independent sample t-test or non-parametric Mann–Whitney test. Any association between season and categorical data was assessed by Chi-square test or Fisher’s exact test. Fisher's exact test was used when the Chi-square test provided unreliable results due to small, expected cell counts (< 5).

Finally, the identification of factors predicting the presence multiple viral, bacterial or viral-bacterial pathogens was assessed using logistic regression models. *P* values ≤ 0.05 were considered statistically significant.

Analysis was performed using IBM SPSS Statistics Version 24.0 (IBM Corp, Armonk, NY, USA).

## Results

### Subject characteristics

Full list of subject characteristics is shown in Supplementary Material (Table 1). Across the two seasons, 2027 individuals were enrolled in the study. Overall, 326 (16.1%) individuals aged 0–4 years, 461 (22.7%) individuals aged 5–17 years, 908 (44.8%) individuals aged 18–64 years and 332 (16.4%) individuals aged ≥ 65 years were enrolled.

1047 (51.7%) were females, 562 (27.7%) had at least one chronic condition and 505 (24.9%) received a flu vaccine during the enrolment season.

### Respiratory infections

The overall incidence of viral and bacterial pathogens is shown in Supplementary Material (Table 2). Influenza A was the most incident viral detection, with A(H3N2) and A(H1N1) with a relative itemset support of 14.40% and 9.91%, respectively. Rhinovirus, coronaviruses and adenovirus were the second, fourth and fifth most common viral infections (14.30%, 5.77% and 5.22%, respectively). Because none of the samples later tested for SARS-CoV-2 yielded a positive result, the incidence rate of SARS-CoV-2 was 0%.

With regards to bacteria, *Haemophilus influenzae*, *Streptococcus pneumoniae* and *Mycoplasma pneumoniae* were the most incident (37.98%, 19.93% and 3.60%, respectively).

Table 1 shows the overall incidence of co-detected viral pairs.

**Table 1:** Incidence of viral pairs in winter seasons 2018/19 and 2019/20

Item set	Itemset size	Itemset support	Relative itemset support (%)
Adenovirus-rhinovirus	2	29	1.43
Enterovirus-rhinovirus	2	17	0.83
Rhinovirus-RSV-A	2	14	0.69
Influenza A(H3N2)-rhinovirus	2	14	0.69
Coronaviruses-rhinovirus	2	12	0.59
Rhinovirus-RSV-B	2	9	0.44
Adenovirus-RSV-A	2	9	0.44
Bocaviruses 1–4-rhinovirus	2	8	0.39
Adenovirus-enterovirus	2	8	0.39
Adenovirus-coronaviruses	2	8	0.39
Metapneumovirus-rhinovirus	2	7	0.34
Influenza A(H3N2)-RSV-B	2	7	0.34
Adenovirus-RSV-B	2	6	0.29
Adenovirus-bocaviruses 1–4	2	5	0.25
Adenovirus-metapneumovirus	2	5	0.25
Enterovirus-RSV-A	2	5	0.25
Coronaviruses-influenza A(H1N1)	2	5	0.25
Coronaviruses-influenza A(H3N2)	2	5	0.25
PIV-3-rhinovirus	2	4	0.20
Coronaviruses-influenza B Victoria	2	4	0.20
Coronaviruses-RSV-A	2	4	0.20
Adenovirus-influenza A(H1N1)	2	4	0.20
PIV-3-RSV-B	2	3	0.15
Adenovirus-PIV-3	2	3	0.15
Enterovirus-bocaviruses 1–4	2	3	0.15
Bocaviruses 1–4-RSV-A	2	3	0.15
Influenza A(H1N1)-metapneumovirus	2	3	0.15
Influenza A(H3N2)-metapneumovirus	2	3	0.15
Enterovirus-RSV-B	2	3	0.15
Adenovirus-influenza A(H3N2)	2	3	0.15

Rhinovirus was the most commonly pathogen found in viral co-detections. Adenovirus-rhinovirus and enterovirus-rhinovirus were in fact the two most incident concomitant viral pairs (1.43% and 0.83%), followed by RSV-A-rhinovirus and influenza A(H3N2)-rhinovirus (0.69% each).

Table 2 shows the overall incidence of co-detected bacterial pairs.

**Table 2:** Incidence of bacterial pairs in winter seasons 2018/19 and 2019/20

Itemset	Itemset size	Itemset support	Relative itemset support (%)
*H. influenzae-S. pneumoniae*	2	276	13.61
*H. influenzae-M. pneumoniae*	2	37	1.82
*M. pneumoniae-S. pneumoniae*	2	20	0.99
*C. pneumoniae-H. influenzae*	2	4	0.20
*B. pertussis-S. pneumoniae*	2	3	0.15
*B. pertussis-H. influenzae*	2	3	0.15
*C. pneumoniae-S. pneumoniae*	2	3	0.15
*B. parapertussis-S. pneumoniae*	2	1	0.05
*B. parapertussis-H. influenzae*	2	1	0.05

*Haemophilus influenzae* and *streptococcus pneumoniae* were the most common pathogens found in bacterial co-detections. Top 3 pairs were *haemophilus influenzae* and *streptococcus pneumoniae* (13.61%), *haemophilus influenzae* and *mycoplasma pneumoniae* (1.82%) and *streptococcus pneumoniae* and *mycoplasma pneumoniae* (0.99%).

Finally, Table 3 shows the overall incidence of viral-bacterial pairs. Top 3 pairs were Rhinovirus and *Haemophilus influenzae* (6.86%), influenza A and *Haemophilus influenzae* (5.28% and 4.29%, respectively for influenza A(H3N2) and A(H1N1)) and rhinovirus and *Streptococcus pneumoniae* (4.00%).

**Table 3:** Incidence of viral-bacterial pairs in winter seasons 2018/19 and 2019/20

Item Set	Itemset size	Itemset support	Relative itemset support (%)
Rhinovirus-*H. influenzae*	2	139	6.86
Influenza A(H3N2)-*H. influenzae*	2	107	5.28
Influenza A(H1N1)-*H. influenzae*	2	87	4.29
Rhinovirus-*S. pneumoniae*	2	81	4.00
Adenovirus-*H. influenzae*	2	73	3.60
Influenza A(H3N2)-*S. pneumoniae*	2	63	3.11
Coronaviruses-*H. influenzae*	2	50	2.47
Influenza A(H1N1)-*S. pneumoniae*	2	50	2.47
Adenovirus-*S. pneumoniae*	2	48	2.37
RSV-A-*H. influenzae*	2	46	2.27
Enterovirus-*H. influenzae*	2	40	1.97
RSV-B-*H. influenzae*	2	39	1.92
Influenza B Victoria-*H. influenzae*	2	38	1.87
RSV-A-*S. pneumoniae*	2	29	1.43
Coronaviruses-*S. pneumoniae*	2	28	1.38
Enterovirus-*S. pneumoniae*	2	24	1.18
Metapneumovirus-*H. influenzae*	2	23	1.13
RSV-B-*S. pneumoniae*	2	20	0.99
Metapneumovirus-*S. pneumoniae*	2	19	0.94
Bocaviruses 1–4-*H. influenzae*	2	18	0.89
Influenza B Victoria-*S. pneumoniae*	2	15	0.74
Bocaviruses 1–4-*S. pneumoniae*	2	14	0.69
PIV-3-*H. influenzae*	2	11	0.54
Influenza B Yamagata-*H. influenzae*	2	9	0.44
Rhinovirus-*M. pneumoniae-*	2	9	0.44
PIV-3-*S. pneumoniae*	2	7	0.34
PIV-4-*H. influenzae*	2	6	0.30
Influenza B Yamagata-*S. pneumoniae*	2	6	0.30
PIV-4-*H. influenzae*	2	5	0.25
Influenza A(H3N2)-*M. pneumoniae*	2	5	0.25

### Correlation between viral and bacterial respiratory pathogens

**Fig. 1:**
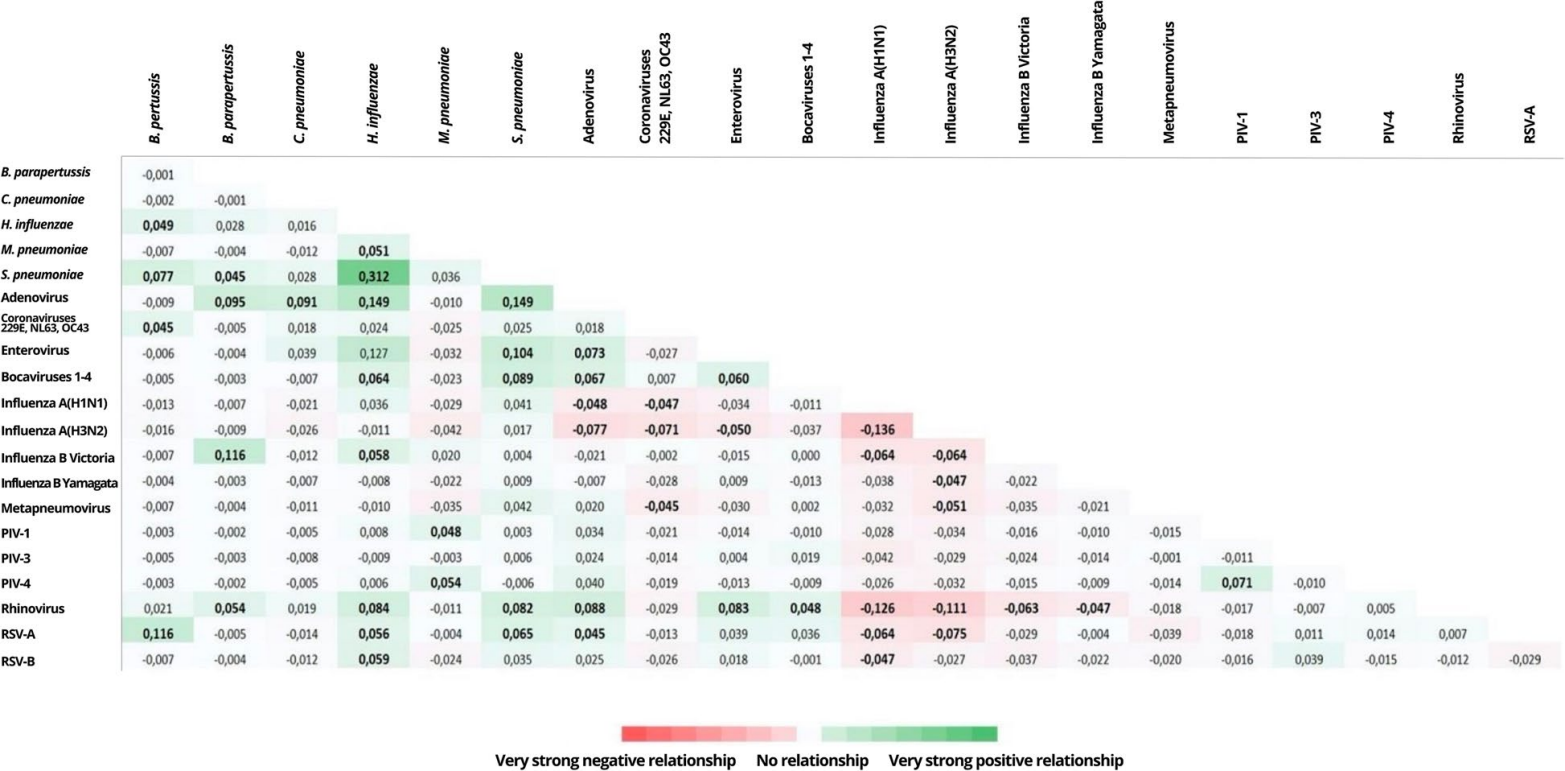
Grade of correlation between different viral and bacterial respiratory pathogens

Figure 1 shows the grade of correlation (in terms of presence) between different viral and bacterial respiratory pathogens.

Overall, there were 31 statistically significant positive associations between pairs of pathogens. The highest strength of effect was the correlation between *Haemophilus influenzae* and *Streptococcus pneumoniae* (Phi = 0.31; *p* < 0.001). The next was between adenovirus and both *Haemophilus influenzae* (Phi = 0.15; *p* < 0.001) and *Streptococcus pneumoniae* (Phi = 0.15; *p* < 0.001). Third, there were the correlations between *Bordetella pertussis* and RSV-A (Phi = 0.12; *p* < 0.001) and *Bordetella parapertussis* and influenza B Victoria (Phi = 0.12; *p* < 0.001).

In addition, there were 18 statistically significant negative associations between pairs of pathogens. The highest strength of effect was shown, among others, for the correlation between influenza A(H1N1) and influenza A(H3N2) (Phi = -0.14; *p* < 0.001), influenza A(H1N1) and rhinovirus (Phi = -0.13; *p* < 0.001), influenza A(H3N2) and rhinovirus (Phi = -0.11; *p* < 0.001), influenza A(H3N2) and RSV-A (Phi = -0.08; *p* = 0.001).

**Fig. 2:**
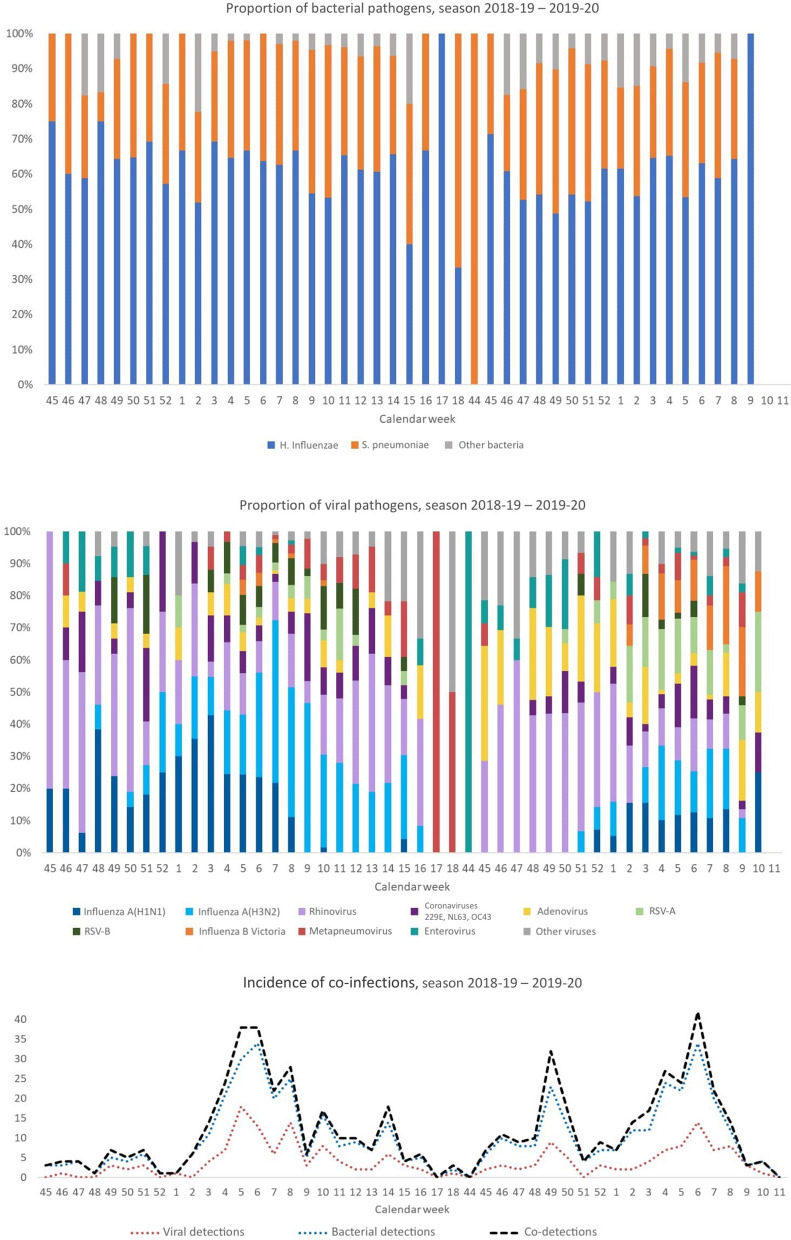
Proportion of the main pathogens of interest (**a**-**b**); incidence of multiple viral, bacterial and viral-bacterial species (**c**)

Circulation by calendar week is illustrated in Fig. 2.

### Characterization of patients detected with single and multiple respiratory pathogens

Factors associated with detection of single pathogens were analyzed and are shown in Supplementary Material (Table 3). The main determinant for the presence of a single pathogen was age. In fact, age groups 5–17, 18–64 and ≥ 65 had, in comparison with the youngest age group 0–4 (ref.), about 2 and 3 times the odds of detection of a single viral or bacterial species. Factors associated with multiple infections are reported in Table 4. The main determinant for the presence of co-detection was also age, but with opposite direction. In fact, all age groups had significant lower odds of co-detection in comparison with the 0–4 age group. With regards to viral co-detection, in particular, the odds were about 64% [OR 0.36 (95%CI 0.24 – 0.55); *p* < 0.001] lower among subjects aged 5–17 years and 94% [OR 0.06 (95%CI 0.03 – 0.12); *p* < 0.001] and 96% [OR 0.04 (95%CI 0.01 – 0.13); *p* < 0.001] lower among adults aged 18–64 years and elderly ≥ 65 years, respectively. With regards to bacterial co-detections, they were 43% [OR 0.57 (95%CI 0.40 – 0.80); *p* = 0.001] lower among subjects aged 5–17 years and 91% [OR 0.09 (95%CI 0.05 – 0.14); *p* < 0.001] and 95% [OR 0.05 (95%CI 0.02 – 0.11); *p* < 0.001] lower among adults aged 18–64 years and elderly ≥ 65 years. As for viruses and bacteria, in comparison with age group 0–4, the odds of co-detection were reduced by 62% [OR 0.38 (95%CI 0.21 – 0.68); *p* = 0.001] among subjects aged 5–17 years and 96% [OR 0.02 (95%CI 0.01 – 0.10); *p* < 0.001] among adults aged 18–64 years.

**Table 4:** Association between demographic characteristics, chronic conditions and risk factors and co-detections

		**Presence of viral co-detections**		**Presence of bacterial co-detections**		**Presence of viral-bacterial co-detections**	
		**Univariate**	**Multivariate**	**Univariate**	**Multivariate**	**Univariate**	**Multivariate**
**Age group**							Ref
	0–4	< 0.001	Ref	< 0.001	Ref	< 0.001	
	5–17		0.36 (0.24 – 0.55); < 0.001		0.57 (0.40 – 0.80); 0.001		0.38 (0.21 – 0.68); 0.001
	18–64		0.06 (0.03 – 0.12); < 0.001		0.09 (0.05 – 0.14); < 0.001		0.02 (0.01 – 0.10); < 0.001
	≥ 65		0.04 (0.01 – 0.13); < 0.001		0.05 (0.02 – 0.11) < 0.001		-
**Males vs females**		0.64	-	0.14	-	0.98	-
**Smoking status**		< 0.001	0.99	< 0.001	0.91	< 0.001	0.99
	Never smoker						
	Former smoker						
	Daily smoker						
	Not reported						
**Season**							-
	2019–2020	0.96	-	0.002	0.07	0.13	
	2018–2019						
**Number of symptoms**	Two or three	< 0.001	0.25	< 0.001	0.91	< 0.001	0.91
	More than three						
**Fever**		< 0.001	0.28	< 0.001	0.99	< 0.001	0.99
**Headache**		< 0.001	0.34	< 0.001	0.16	< 0.001	0.49 (0.26 – 0.96); 0.036
**Myalgia**		< 0.001	0.87	< 0.001	0.49	< 0.001	0.74
**Malaise**		< 0.001	0.80	0.001	0.52	0.001	0.55
**Cough**		0.029	2.10 (1.14 – 3.89); 0.018	0.97	-	0.05	0.09
**Difficulty breathing**		0.44	-	0.64	-	0.24	-
**Sore throat**		< 0.001	0.56	< 0.001	0.67 (0.49 – 0.91); 0.010	< 0.001	0.34
**Influenza vaccination in current season**		0.77	-	0.24	-	0.93	-
**Presence of at least one chronic disease**		< 0.001	0.87	< 0.001	0.30	< 0.001	0.16
**Antiviral treatment within the 2 weeks before swab**		0.30	-	0.99	-	0.99	-
**Statin use at the time of vaccination**		0.020	0.72	0.003	0.98	0.99	-

Our results also indicate differential clinical features between the presence of viral and bacterial pathogens.

Particularly, individuals with both single viral detection and viral-viral detection had higher odds of reporting cough. The odds, however, were higher in case of viral-viral detection [OR 2.10 (95%CI 1.14 – 3.89); *p* = 0.018 vs. OR 1.81 (95%CI 1.30 – 2.52); *p* < 0.001]. On the other hand, individuals detected with a single bacterial species had 39% lower odds of cough [OR 0.61 (95%CI 0.42–0.87); *p* = 0.007]. This association was not shown in case of bacterial co-detection for which, however, the odds of reporting a sore throat were 33% lower [OR 0.67 (95%CI 0.49 – 0.91); *p* = 0.010].

Finally, individuals with a viral-bacterial co-detection had, in comparison with other patients, 51% lower odds of reporting headache [OR 0.49 (95%CI 0.26- 0.96); *p* = 0.036].

## Discussion and conclusions

In this study, the most common viral and bacterial pathogens found in respiratory samples were, respectively, influenza A and rhinovirus and *Haemophilus influenzae* and *Streptococcus pneumoniae*.

Rhinovirus, in particular, was the most present in viral co-detections, frequently in pair with adenovirus, enterovirus, RSV-A or influenza A(H3N2).

*Haemophilus influenzae* and *Streptococcus pneumoniae* were instead the most present in bacterial co-detections, in pair with each other or *Mycoplasma pneumoniae.*

Finally, the top 3 viral-bacterial pairs were rhinovirus*-Haemophilus influenzae*, influenza A-*Haemophilus influenzae* and rhinovirus*-Streptococcus pneumoniae*.

The highest strength of correlation was found for bacterial-bacterial or viral-bacterial pairs such as *Haemophilus influenzae-Streptococcus pneumoniae,* adenovirus*-Haemophilus influenzae*, adenovirus*-Streptococcus pneumoniae*, RSV-A-*Bordetella pertussis* and influenza B Victoria*-Bordetella parapertussis*.

By contrast, viral-viral pairs were detected together at significant lower rates than bacterial-bacterial or viral-bacterial pairs. Rhinovirus, influenza, and RSV, in particular, showed a significant negative correlation between each other, with the highest negative values found for the pairs influenza A(H1N1)-influenza A(H3N2), rhinovirus-influenza and RSV-influenza.

These results align with prior analyses, revealing that co-detections of common respiratory viruses often occur at considerably lower rates than would be expected by chance alone [11–14]. These observations support the viral interference hypothesis, according to which previous exposure of cells to another virus inhibits viral reproduction. Factors implicated are the generation of interferons by infected cells and the occupation or down-modulation of cellular receptors [11].

On the other hand, potentially pathogenic bacteria tend to colonize the upper airways, with a proven increase in density and frequency of colonization during viral infections [15]. In vitro studies have shown that influenza and RSV viruses have the ability to augment bacterial adherence to the respiratory epithelium by up-regulating cell receptors [16]. Recent advances in microbiome research indicate that the mechanisms by which respiratory viruses promote bacterial infections are diverse and go from damaging the airways to dysregulating the immune responses and can even involve the mediated release of bacteria from biofilms [17].

In agreement with other studies we found that co-detection is substantially more common in children, especially under the age of 5 [18–20].

With regards to signs and symptoms, our results substantiate with prior literature suggesting that cough is a valid indicator of viral co-detections [21]*.* However, it's worth acknowledging that certain observed associations may be biased. For instance, the observed diminished odds of cough and sore throat among individuals detected with bacterial species and headache among individuals detected with viruses and bacteria, could be attributed to the self-reporting of symptoms. Indeed, our analysis has demonstrated a significant increase in co-detection rates among young children aged 0 to 4 years, that may not possess the ability to effectively communicate subjective symptoms like "sore throat" and "headache," but can still exhibit the action of coughing.

In addition, we were unable to differentiate between different types of cough (eg. dry, typically viral, versus productive, typically bacterial).

Our study presents with additional limitations. In fact, the evaluation of co-detections was not one of the initial outcomes of the DRIVE study, which incapacitated us from following up patients over time and potentially analyze the sequence of additional infections.

The determinants for adjustment in the multivariable analysis were also chosen on the basis of another outcome (namely, influenza vaccine effectiveness). Thus, it is possible that other determinants relevant to our research question were overlooked.

Finally, because all of our samples came from individuals undergoing outpatient visits for ILI and none of them were hospitalized, we were not able to analyse different clinical outcomes among co-detection groups. Nevertheless, other studies have found that morbidity and mortality are significantly higher in patients with viral-bacterial co-infections in comparison with individuals detected with a single viral or bacterial species [5, 22], underlying the need for correct and punctual information on the relationship between respiratory pathogens.

A significant strength of our study lies in the use of the *apriori* algorithm, usually employed in other domains such as market basket analyses, to evaluate the real incidence of respiratory pathogens, alone and in combination. In fact, the algorithm was initially developed for the field of retail and consumer behavior, where it identifies frequent itemsets (combinations of items frequently bought together). In the context of respiratory pathogens, the algorithm helps uncover frequent co-occurrences, revealing hidden patterns and associations among pathogens that might not be evident through traditional methods.

In our study, we indeed observed that certain pathogens appeared together more frequently, suggesting potential synergistic relationships, and certain pathogens appeared together less frequently, suggesting potential antagonistic relationships.

The subsequent correlation analysis solidified these observations, providing evidence of the existence of both positive and negative correlations between the investigated pathogens.

Another advantage to our study is that we were able to test the presence of a high number of respiratory pathogens (19 viruses and 7 bacteria) in a similar sample of individuals living in the same area and presenting with ILI symptoms, for a total of 2027 individuals with an oropharyngeal swab.

Finally, none of our samples later tested for SARS-CoV-2 yielded a positive result. However, given the change in the epidemiological panorama following the spread of the COVID-19 pandemic, future studies employing the methodology here described and taking in account the circulation of SARS-CoV-2 could further enrich the body of evidence on the concurrency of respiratory pathogens.

### Supplementary Information

Supplementary Material 1.

## Data Availability

Further details and results from the DRIVE studies are available at the DRIVE website via the following link: https://www.drive-eu.org/ (accessed on 14 November 2022).
