# Supplementary material for: Co-detection of respiratory pathogens among ILI patients: characterization of samples collected during the 2018/19 and 2019/20 pre-pandemic seasons
Source: BMC Infect Dis. 2024 Aug 29;24:881. doi: 10.1186/s12879-024-09687-1 (PMC11361097; doi:10.1186/s12879-024-09687-1)
Supplement: Supplementary file 1 — Supplementary Material 1. [file 12879_2024_9687_MOESM1_ESM.docx]

| **Supplementary Material**  **Table 1 - Subject characteristics** | | | | | |
| --- | --- | --- | --- | --- | --- |
|  |  | **Total** | **2018-2019** | **2019-2020** | **p** |
| Sex | Females | 1047 (51.7%) | 574 (51.3%) | 473 (52.1%) | 0.72 |
|  | Males | 980 (48.3%) | 545 (48.7%) | 435 (47.9%) |  |
| Age | | 34.0 ± 26.22 | 36.1 ± 26.23 | 31.5 ± 26.01 | <0.001* |
| BMI | | 25.2 ± 5.30 | 24.2 ± 4.49 | 25.6 ± 5.63 | 0.23 |
| Smoking status | Not reported | 30 (1.5%) | 11 (1.0%) | 19 (2.1%) | 0.15 |
|  | Never smoker | 1548 (76.4%) | 835 (74.6%) | 713 (78.5%) |  |
|  | Former smoker | 250 (12.3%) | 153 (13.7%) | 97 (10.7%) |  |
|  | Daily smoker | 199 (9.8%) | 120 (10.7%) | 79 (8.7%) |  |
| Influenza vaccination in current season | No | 1522 (75.1%) | 838 (74.9%) | 684 (75.3%) | 0.82 |
|  | Yes | 505 (24.9%) | 281 (25.1%) | 224 (24.7%) |  |
| Vaccine brand | Agrippal | 24 (4.8%) | 14 (5.0%) | 10 (4.5%) |  |
|  | Fluad | 63 (12.5%) | 27 (9.6%) | 36 (16.1%) |  |
|  | Fluarix Tetra | 384 (76.0%) | 237 (84.3%) | 147 (65.6%) |  |
|  | Flucelvax Tetra | 14 (2.8%) | 0 (0.0%) | 14 (6.3%) |  |
|  | Influvac Tetra | 1 (0.2%) | 1 (0.4%) | 0 (0.0%) |  |
|  | Vaxigrip Tetra | 19 (3.8%) | 2 (0.7%) | 17 (7.6%) |  |
| Statin use at the time of vaccination | No | 1927 (95.1%) | 1069 (95.5%) | 858 (94.5%) | 0.28 |
|  | Yes | 100 (4.9%) | 50 (4.5%) | 50 (5.5%) |  |
| Antiviral treatment within the 2 weeks before swabbin | No | 2023 (99.8%) | 1116 (99.7%) | 907 (99.9%) | 0.63 |
|  | Yes | 4 (0.2%) | 3 (0.3%) | 1 (0.1%) |  |
| Presence of at least one chronic disease | No | 1465 (72.3%) | 796 (71.1%) | 669 (73.7%) | 0.20 |
|  | Yes | 562 (27.7%) | 323 (28.9%) | 239 (26.3%) |  |
| Number of symptoms | Two or three | 338 (16.7%) | 157 (14.0%) | 181 (19.9%) | <0.001* |
|  | More than three | 1689 (83.3%) | 962 (86.0%) | 727 (80.1%) |  |
| Fever | No | 224 (11.1%) | 130 (11.6%) | 94 (10.4%) | 0.37 |
|  | Yes | 1803 (88.9%) | 989 (88.4%) | 814 (89.6%) |  |
| Headache | No | 868 (42.8%) | 453 (40.5%) | 415 (45.7%) | 0.018* |
|  | Yes | 1159 (57.2%) | 666 (59.5%) | 493 (54.3%) |  |
| Myalgia | No | 832 (41.0%) | 407 (36.4%) | 425 (46.8%) | <0.001* |
|  | Yes | 1195 (59.0%) | 712 (63.6%) | 483 (53.2%) |  |
| Malaise | No | 261 (12.9%) | 129 (11.5%) | 132 (14.5%) | 0.044* |
|  | Yes | 1766 (87.1%) | 990 (88.5%) | 776 (85.5%) |  |
| Cough | No | 270 (13.3%) | 140 (12.5%) | 130 (14.3%) | 0.23 |
|  | Yes | 1757 (86.7%) | 979 (87.5%) | 778 (85.7%) |  |
| Difficulty breathing | No | 1749 (86.3%) | 962 (86.0%) | 787 (86.7%) | 0.65 |
|  | Yes | 278 (13.7%) | 157 (14.0%) | 121 (13.3%) |  |
| Sore throat | No | 498 (24.6%) | 278 (24.8%) | 220 (24.2%) | 0.75 |
|  | Yes | 1529 (75.4%) | 841 (75.2%) | 688 (75.8%) |  |
| Number of comorbidities | No comorbidities | 1465 (72.3%) | 796 (71.1%) | 669 (73.7%) | 0.63 |
|  | One | 367 (18.1%) | 209 (18.7%) | 158 (17.4%) |  |
|  | Two | 121 (6.0%) | 71 (6.3%) | 50 (5.5%) |  |
|  | Three or more | 74 (3.7%) | 43 (3.8%) | 31 (3.4%) |  |
| Chronic liver disease | No | 2021 (99.7%) | 1114 (99.6%) | 907 (99.9%) | 0.17 |
|  | Yes | 6 (0.3%) | 5 (0.4%) | 1 (0.1%) |  |
| Diabetes | No | 1929 (95.2%) | 1062 (94.9%) | 867 (95.5%) | 0.55 |
|  | Yes | 98 (4.8%) | 57 (5.1%) | 41 (4.5%) |  |
| Cardiovascular diseases | No | 1703 (84.0%) | 933 (83.4%) | 770 (84.8%) | 0.38 |
|  | Yes | 324 (16.0%) | 186 (16.6%) | 138 (15.2%) |  |
| Cancer | No | 1972 (97.3%) | 1089 (97.3%) | 883 (97.2%) | 0.92 |
|  | Yes | 55 (2.7%) | 30 (2.7%) | 25 (2.8%) |  |
| Immunodeficiency or organ transplant | No | 2019 (99.6%) | 1113 (99.5%) | 906 (99.8%) | 0.26 |
|  | Yes | 8 (0.4%) | 6 (0.5%) | 2 (0.2%) |  |
| Lung disease | No | 1781 (87.9%) | 979 (87.5%) | 802 (88.3%) | 0.57 |
|  | Yes | 246 (12.1%) | 140 (12.5%) | 106 (11.7%) |  |
| Anemia | No | 2016 (99.5%) | 1111 (99.3%) | 905 (99.7%) | 0.24 |
|  | Yes | 11 (0.5%) | 8 (0.7%) | 3 (0.3%) |  |
| Renal disease | No | 2002 (98.8%) | 1102 (98.5%) | 900 (99.1%) | 0.20 |
|  | Yes | 25 (1.2%) | 17 (1.5%) | 8 (0.9%) |  |
| Dementia | No | 2020 (99.7%) | 1116 (99.7%) | 904 (99.6%) | 0.51 |
|  | Yes | 7 (0.3%) | 3 (0.3%) | 4 (0.4%) |  |
| History of stroke | No | 2010 (99.2%) | 1113 (99.5%) | 897 (98.8%) | 0.10 |
|  | Yes | 17 (0.8%) | 6 (0.5%) | 11 (1.2%) |  |
| Rheumatologic diseases | No | 2006 (99.0%) | 1110 (99.2%) | 896 (98.7%) | 0.25 |
|  | Yes | 21 (1.0%) | 9 (0.8%) | 12 (1.3%) |  |
| Obesity | No | 1978 (97.6%) | 1087 (97.1%) | 891 (98.1%) | 0.15 |
|  | Yes | 49 (2.4%) | 32 (2.9%) | 17 (1.9%) |  |

**Table 2 - Overall incidence of viral and bacterial respiratory pathogens estimated by employing the *apriori* algorithm**

| **Itemset** | **Itemset Size** | **Itemset Support** | **Relative Itemset Support (%)** |
| --- | --- | --- | --- |
| **Viral infections** | | | |
| Influenza A(H3N2) | 1 | 292 | 14.40 |
| Rhinovirus | 1 | 290 | 14.30 |
| Influenza A(H1N1) | 1 | 201 | 9.91 |
| Coronaviruses | 1 | 117 | 5.77 |
| Adenovirus | 1 | 106 | 5.22 |
| RSV-A | 1 | 91 | 4.48 |
| RSV-B | 1 | 74 | 3.65 |
| Influenza B Victoria | 1 | 72 | 3.55 |
| Metapneumovirus | 1 | 65 | 3.20 |
| Enterovirus | 1 | 53 | 2.61 |
| PIV-3 | 1 | 32 | 1.57 |
| Bocaviruses 1-4 | 1 | 28 | 1.38 |
| Influenza B Yamagata | 1 | 26 | 1.28 |
| PIV-1 | 1 | 14 | 0.69 |
| PIV-4 | 1 | 12 | 0.59 |
| **Bacterial infections** | | | |
| *H. influenzae* | 1 | 770 | 37.98 |
| *S. pneumoniae* | 1 | 404 | 19.93 |
| *M. pneumoniae* | 1 | 73 | 3.60 |
| *C. pneumoniae* | 1 | 8 | 0.39 |
| *B. pertussis* | 1 | 3 | 0.14 |
| *B. parapertussis* | 1 | 1 | 0.04 |

**Table 3 - Association between demographic characteristics, chronic conditions and risk factors and the presence of single pathogen infections**

|  |  | **Presence of single viral infection** | | **Presence of single bacterial infection** | |
| --- | --- | --- | --- | --- | --- |
|  |  | **Univariate** | **Multivariate** | **Univariate** | **Multivariate** |
| **Age group** |  |  |  |  | Ref |
|  | 0-4 | <0.001 | Ref | <0.001 |  |
|  | 5-17 |  | 1.79 (1.19 – 2.69); 0.005 |  | 0.10 |
|  | 18-64 |  | 2.98 (2.05 – 4.35); <0.001 |  | 2.39 (1.39 – 4.11); 0.002 |
|  | ≥ 65 |  | 2.76 (1.76 – 4.33); <0.001 |  | 2.97 (1.56 – 5.65); 0.001 |
| **Males vs females** |  | 0.003 | 0.79 (0.65 – 0.96); 0.018 | 0.67 | - |
| **Smoking status** |  | 0.07 | - | 0.045 | 0.30 |
|  | Never smoker |  |  |  |  |
|  | Former smoker |  |  |  |  |
|  | Daily smoker |  |  |  |  |
|  | Not reported |  |  |  |  |
| **Season** |  |  |  |  |  |
|  | 2019-2020 | <0.001 | Ref. | 0.78 | - |
|  | 2018-2019 |  | 1.34 (1.10 – 1.64); 0.004 |  |  |
| **Number of symptoms** |  |  |  |  |  |
|  | Two or three | <0.001 | 0.97 | 0.54 | - |
|  | More than three |  |  |  |  |
| **Fever** |  | 0.54 | - | 0.039 | 0.31 |
| **Headache** |  | <0.001 | 0.46 | 0.16 | - |
| **Myalgia** |  | <0.001 | 1.32 (1.04 – 1.68); 0.024 | 0.021 | 0.25 |
| **Malaise** |  | 0.021 | 0.56 | 0.70 | - |
| **Cough** |  | <0.001 | 1.81 (1.30 – 2.52); <0.001 | 0.018 | 0.61 (0.42 – 0.87); 0.007 |
| **Difficulty breathing** |  | 0.31 | - | 0.84 | - |
| **Sore throat** |  | 0.33 | - | 0.12 | - |
| **Influenza vaccination**  **in current season** |  | 0.35 | - | 0.026 | 0.20 |
| **Presence of at**  **least one**  **chronic disease** |  | <0.001 | 0.28 | 0.021 | 0.62 |
| **Antiviral treatment within the 2 weeks before swab** |  | 0.99 | - | 0.99 | - |
| **Statin use at the time of vaccination** |  | 0.06 | - | 0.24 | - |
